# Reassessment of the dementia diagnosis of Alzheimer's disease in patients enrolled on the cholinesterase inhibitors dispensation program

**DOI:** 10.1590/S1980-57642012DN06040012

**Published:** 2012

**Authors:** Magda Cristina Flaitt Sanches Piovesana, Fúlvio Rogério Garcia, Kátia G. Carrasco, Waldir Antonio Tognola

**Affiliations:** 1 Enfermeira, Mestre em Medicina e Ciências da Saúde, Faculdade de Medicina de São José do Rio Preto, SP, Brazil.; 2 Médico Contratado, Faculdade de Medicina de São José do Rio Preto, SP, Brazil.; 3 Psicóloga, Mestranda em Medicina e Ciências da Saúde, Faculdade de Medicina de São José do Rio Preto, SP, Brazil.; 4 Professor emérito da Faculdade de Medicina de São José do Rio Preto, SP, Brazil.

**Keywords:** Alzheimer's disease, anti-cholinesterases, diagnosis assessment

## Abstract

**Objective:**

Reassess the diagnosis of Alzheimer's Disease (AD) in patients treated with anti-cholinesterases dispensed by High Cost Drug stores (Exceptional Drugs Program).

**Methods:**

A prospective study to reassess the diagnosis of probable Alzheimer's Disease was conducted (AD). The patients were submitted to the protocol of dementia investigation at the Neurogeriatric Outpatient Clinic of the Teaching Hospital de Base de São José do Rio Preto. Groups were classified using the criteria of the National Institute of Neurologic and Communicative Diseases and Vascular Cerebral Accident and Alzheimer Disease Related Association (NINCDS-ADRDA). The study was completed by applying the Disability Assessment for Dementia (DAD). The significance level was set at 5%.

**Results:**

106 patients participated, selected randomly from a group of 390 patients contacted when receiving their medication at the High Cost Drug store. Two groups were formed: the first, containing 52 patients who fulfilled criteria for AD (FC Group); and a second, with 54 patients not fulfilling criteria (NFC). The FC Group had older age, worse performance on the Mini-Mental State Exam (MMSE) and poorer performance on the DAD. Also, treatment time was longer and drugs doses higher in the FC Group.

**Conclusion:**

Study results showed a high number of patients using anti-cholinesterases that did not fulfill the diagnosis criteria for probable AD. Comparison of the two groups revealed different behavior between them, corroborating the hypothesis of inadequate inclusion of the NFC Group patients in the Exceptional Drugs Program.

## Abstract (pt)

**Objetivo:**

Reavaliar o diagnóstico de Doença de Alzheimer (DA) nos pacientes em tratamento com anticolines-terásicos dispensados pelas farmácias de alto custo (Programa de Medicamentos Excepcionais).

**Métodos:**

Estudo prospectivo, para reavaliação do diagnóstico de Doença de Alzheimer (DA) provável. Os pacientes foram submetidos ao protocolo de investigação de demências do ambulatório de Neurogeriatria do Hospital de Base de São José do Rio Preto. Utilizou-se para classificação nos grupos critérios do Instituto Nacional de Doenças Neurológicas e Comunicativas e Acidente Vascular Cerebral e Associação da Doença de Alzheimer e Doenças Relacionadas (NINCDS-ADRA). Completou-se o estudo utilizando-se a Escala para Avaliação de Incapacidades na Demência (DAD). Foi estabelecido o nível de significância em 5%.

**Resultados:**

Participaram 106 pacientes, selecionados aleatoriamente de um grupo de 390 pacientes contactados quando recebiam o medicamento na farmácia de alto-custo. Obtiveram-se dois grupos: o primeiro que preencheu critérios para DA (Grupo PC), que incluiu 52 pacientes e o segundo grupo o qual não preencheu critérios (Grupo NPC) com 54 pacientes. O Grupo PC apresentou: idade mais elevada, pior performance no Mini Exame do Estado Mental (MEEM) e desempenho inferior na DAD. O tempo de tratamento era maior e doses mais elevadas dos medicamentos no Grupo PC.

**Conclusão:**

De acordo com os resultados do estudo, observou-se um grande número de pacientes utilizando anticolinesterásicos que não preencheram os critérios diagnósticos para DA provável. Na comparação dos dois grupos observou-se comportamento diferente dos mesmos, que permitiram corroborar com a hipótese de inclusão inadequada dos pacientes do Grupo NPC no Programa de Medicamentos Excepcionais.

## INTRODUCTION

The growth of the elderly population in clearly one of the most noteworthy current phenomena, with varied and intricate social consequences. This shift fosters the incidence and prevalence of degenerative chronic diseases associated to aging, among which include those causing dementia.^1,2^ Epidemiologic studies indicate a high prevalence of dementia in elderly people, representing unequivocal data proving that age is a significant risk factor.^3^

Alzheimer's Disease (AD) is the main cause of cognitive decline in the elderly, representing more than half of all dementia cases.^4^ In a population-based study carried out in Catanduva city, in São Paulo State (SP), a 7.1% prevalence of dementia cases was observed in individuals aged 65 years or older with AD being the most frequent etiology, found in 55.1% of the dementia cases.^5^

There is currently no etiologic treatment for AD available, with treatment comprising pharmacological strategies based on the presupposition of cholinergic deficit.^4^ The main aim is to regulate this in the central nervous system with cholinesterase inhibitors (I-ChE).^6^ The Brazilian Academy of Neurology recommends treatment with cholinesterase inhibitors as effective for Alzheimer's disease.^7^ Currently, three I-ChEs are fully approved for clinical use: Rivastigmine, Galantamine and Donepezil.^8^ In the medical literature there are a number of studies demonstrating the efficiency of cholinesterase inhibitors in AD,^9-11^ hence the government program which makes this medication available to the population.

Ensuring access to medications is a complementary and essential part of an adequate health care policy. The Unified Health System (SUS) has been engaged in providing free high-cost medications. These drugs, also called "exceptional" are included under the Exceptional Drugs Program.^12^

In order to provide the population with treatment, the Ministry of Health established the "Assistance Program for People with Alzheimer's disease", the Protocol Clinical Guideline for the treatment of Alzheimer's disease, included the cholinesterase inhibitors Rivastig-mine, Galantamine and Donepezil in the list of high-cost medications, and determined criteria for inclusion/ exclusion of patients for treatment, diagnostic criteria, recommended a therapeutic regimen and mechanisms for monitoring and evaluating treatment.^13^

For inclusion in this protocol patients must: provide an assessment of a neurologist and/or psychiatrist and/ or geriatrician; fulfill the clinical criteria of dementia due to possible or probable Alzheimer's Disease, have undergone the Mini Mental State Exam (MMSE) with a score of between 12 and 24 for patients schooled beyond primary education, and between 8 and 17 for those with up to primary education; Individuals with a Clinical Dementia Rating (CDR) of 1 or 2 (moderate or mild dementia) will not be included in this Treatment protocol nor will patients exhibiting at least one of the following: poor adherence to the treatment, evidence of non-compensated simultaneous organic or metabolic cerebral lesions, severe cardiac insufficiency or cardiac arrhythmia, parkinsonian syndrome, diarrhea, and peptic disease without response to treatment.^8^

As criteria to discontinue the treatment, the following variables will be checked: absence of improvement or steady deterioration of the patient's clinical picture on reassessment three or four months after the beginning of treatment; even for those on continued treatment, treatment must only be maintained while the MMSE score remains above 12 (twelve) for patients with greater than primary education and above 8 (eight) for patients with incomplete primary education, as there has been no evidence of treatment benefit with fewer years of education and thus treatment should be suspended.^8^

Due to the social relevance of AD, halting disease evolution is a major priority. The Exceptional Drugs Program makes the anti cholinesterases available for use in the initial and intermediate phases of the disease. No publications evaluating the effectiveness of the anti cholinesterases dispensation program are available. Evidence from clinical practice has shown that some patients using the drugs did not fulfill the diagnosis criteria for AD, generating unnecessary cost.

The aim of this study was to reassess the diagnosis of patients under the Exceptional Drugs Program and to demonstrate that the group with confirmed AD differed to the group without AD.

## METHODS

The study was performed at the Neurogeriatric Outpatient Clinic, of the Medical School of São José do Rio Preto - FAMERP and Hospital de Base.

Patients living in the municipality of São José do Rio Preto, SP - Brazil were included in the study. They were enrolled in the "Assistance Program for Alzheimer's Disease Carriers"; supported by the service to provide medication of specialized composition from the Pharmaceutical Attendance Program (High Cost);^14^ and in use of anti cholinesterases for at least three months. All subjects signed the free informed consent form after agreeing to participate in this scientific investigation.

In order to reassess the diagnosis of probable AD, the following steps were followed:

**PHASE I - Sample selection.** In this prospective study, the sample selection occurred in the period between May 2008 and February 2010. During this time, the High Cost Drug stores attended an average of 835 patients a month. During the data collection, of the 390 patients who were initially contacted, only 106 patients participated in all the phases of the study.

Patients were invited to participate in the study during the monthly dispensing of the drug at the High Cost Drug stores, and in most cases the patients were represented by a family member.

**PHASE II - Diagnosis reassessment.** The patients were submitted to the dementia protocol of the Neurogeriatric Outpatient Clinic of Hospital de Base, which includes clinical assessment, review of the Mini-Mental State Exam (MMSE),^15,16^ laboratory and imaging exams, following IDC-10^17^ considering those who already had exams.

To certify the AD diagnosis, patients were assessed by two neurologists and a psychologist, using the National Institute of Neurologic and Communicative Diseases and Vascular Cerebral Accident (NINCDS) and Alzheimer Disease Related Association (ADRDA)^18^ criteria, which allows the classification of dementia of the Alzheimer type (AD) into three different levels: definitive AD - requires the presence of probable or possible AD with neuropathological confirmation; probable AD - this is the chosen diagnosis when the dementia syndrome has slow and progressive onset and when other etiological factors have been excluded; possible AD -the available alternative to classify the disease when the clinical picture is atypical or there is a concomitant physical condition which may also cause dementia.

After this analysis, patients were categorized into two groups, those who fulfilled the criteria (FC Group), and those who did not fulfill the diagnostic criteria (NFC Group) for AD. The group that did not fulfill the criteria was not studied further and included several clinical situations.

**PHASE III - Group analysis.** FC and NFC Groups were compared to better reassess the AD diagnosis. This was done based on Sociodemographic characteristics such as age, sex, education as well as Clinical characteristics: MMSE values, treatment duration, prescribed types and doses of anti-cholinesterases, plus a scale for the Disability of Assessment for Dementia (DAD) which evaluates basic, instrumental and leisure activities. The total score is 100 and lower values denote a higher hindrance in activities of daily living^19-23^.

Due to ethical reasons, those who took the patient to the Exceptional Drugs Program have not been identified, and the study results have been presented to the program manager in a general sense.

**Statistical analysis.** The statistical analysis was performed by means of tables of crossed frequencies to assess association between the group that fulfilled the criteria and the group that did not fulfill criteria for Alzheimer's disease, according to the classified qualitative or quantitative variables.

The Chi-square test was used to measure the association of categorical variables (gender, age group, educational level, length of treatment, anticholines-terase prescribed, MMSE, DAD) along with Fisher's exact test.

The medians were submitted to the Kruskal-Wallis test to determine the level of statistical significance. The Student's *t*-test was used to compare the variables (age and age versus gender).

The level of significance was set at 5% (p<0.05).

**Ethical approach.** This study was approved by the Human Research Ethics Committee under protocol #2644/2008.

## RESULTS

One hundred and six patients participated in the study. Subjects were classified into either the FC Group, when they fulfilled the criteria for AD, or into the NFC Group when they did not. According to the method used, 52 (49%) patients were classified as probable AD.

**Socio-demographic characteristics of the groups.** In the FC Group, there was a predominance of women: 39 women (75%) and 13 men (25%) whereas in the NFC Group, there were 29 women (54%) and 25 men (46%) (Table 1).

**Table 1:** Distribution of patients by age, education, and gender according to fulfillment (FC) or non-fulfillment (NFC) of Alzheimer's Disease (AD) criteria.

	n	Age in years (mean)	Education up to 3 years	Gender (%)	
				**Male**	**Female**
FC Group	52	77.8	77.0	25	75
NFC Group	54	74.2	68.0	46	54

Age in the FC Group ranged from 60 to 94 years (mean=77.8 years old, standard deviation (sd)=7. 95, mode (m)=78 years). In the NFC Group, age ranged from 52 to 88 years (mean=74.2 years old, sd=7.74, m =74 years). The statistical analysis showed that the mean age of those who did not have confirmed AD was lower than the group that had confirmed AD (t-Test=0; p=0.019) (Table 1).

Analysis of both FC and NFC groups according to sex and age revealed no significant evidence of differences in mean age and sex of patients with AD confirmation p=0.278).

In the FC Group , 41 patients (77%) had less than three years of education. In the NFC Group , 37 (68%) had education of up to 3 years. The statistical analysis showed that the variable of educational level had no positive association between the FC and NFC Groups for AD. (Chi-Square=5.900; p=0.207) (Table 1).

**Clinical characteristics.** It was observed that 44 (85%) patients from the FC Group had low MMSE while in the NFC Group 27 (50%) had normal values and 27 (50%) low scores. Although this group of patients showed cognitive deficit, it was not characterized as AD. The analysis showed evidence of a positive association between low MMSE and AD confirmation (Chi-Square =14.35; p=0.000).

All patients of the FC Group had been undergoing treatment with anti-cholinesterases for more than six months, and 33% had been undergoing the treatment for between 13 and 24 months. In the NFC Group, 13% of patients had been on anticholinesterase treatment for less than 6 months whereas 41% had been receiving anticholinesterase for between 6 and 12 months. A correlation was observed between longer time on treatment and confirmation of AD diagnosis (Chi-Square: 20.009) (Table 2).

**Table 2:** Comparison of groups according to AD treatment duration.

Treatment duration (months)	FC Group			NFC Group		p
	**n**	**%**		**n**	**%**	
						0.001
<6	0	0		7	12.9	
6 to 12	9	17.3		22	40.7	
13 to 24	17	32.6		12	22.2	
25 to 36	9	17.3		8	14.8	
37 to 48	5	9.6		1	1.8	
49 or +	12	23.0		4	7.4	

In the present study, the pharmacological interventions for AD were based on the three available cholin-esterase inhibitors: Rivastigmine, Galantamine and Donepezil. In the FC Group, 44% of patients used Riv-astigmine, 44% Galantamine and 11% Donepezil. It was observed that the medications were prescribed in effective or full doses. For the NFC Group , 59% of patients used Rivastigmine, 35% Galantamine and 5% Done-pezil. It is noteworthy that the lowest doses of 1.5 mg-Rivastigmine were being administered to 18 patients (33%). (Chi Square =13. 5 and p=0.13) (Table 3).

**Table 3:** Pharmacological treatment with anticholinesterases, according to drug type and dose in Groups (FC and NFC).

Drug	Dose	FC Group %	NFC Group %	p
				0.13
Rivastigmine	1.5 mg	7.6	33.3	
Rivastigmine	2 mg	1.9	5.5	
Rivastigmine	3 mg	15.3	9.2	
Rivastigmine	4.5 mg	9.6	5.5	
Rivastigmine	6 mg	9.6	5.5	
Galantamine	8 mg	11.5	12.9	
Galantamine	16 mg	15.3	9.2	
Galantamine	24 mg	17.3	12.9	
Donepezil	5 mg	7.6	3.7	
Donepezil	10 mg	3.8	1.85	

On the evaluation according to the DAD scale, whose results ranged from zero percent (greater functional dependence) to a hundred percent (greater functional independence); the median score of the FC Group was 25.5 (mean=29.7, sd=25.9, m=5). In the NFC Group, the median DAD score was 76.5 (mean=66.1, sd =35.34, m=100). The statistical analysis showed evidence of the median for performance of daily activities, i.e. this value was lower in the confirmed AD group. (Chi-square: 21.74; p=0.000 and CI=95% (-76.0;-37.0) (Table 4).

**Table 4:** Comparison between FC and NFC Groups, according to performance on DAD.

DAD	FC Group			NFC Group		p
	**%**	**Median**		**%**	**Median**	
		25.5			76.5	0
50 to100%	24.8			72.1		
0 to 49%	75.0			27.6		

## DISCUSSION

AD is an important social economic problem, and the government has established the dispensation of medications for its treatment. However, it has been noted that many patients who were taking the anti-cholinesterases did not fulfill the diagnostic criteria, implying an unnecessary cost since these are high cost medications. The evidence in scientific publications points to the effectiveness of the treatment, but there is a lack of studies analyzing diagnosis reassessment.

Application of the protocol and confirmation criteria for AD resulted in 2 groups of patients: those who fulfilled the criteria and those who did not. The NFC Group was heterogeneous with several diagnoses, including age-related cognitive deficit, vascular dementia, depression, or other psychiatric and clinical manifestations, where this group was not relevant to the aim of this study. However, comparison of both groups seemed appropriate, for they originally formed the same group.

Forty-nine percent of patients had probable AD diagnosis, and the remaining 51% did not fulfill the criteria for AD. Anunciação et al. 24 found similar results in a study performed in Rio de Janeiro. They showed that only 50% of the patients diagnosed with AD fulfilled fully integrated requirements of ICD-10, thus confirming the great difficulty in establishing the diagnosis for AD.^5^

Comparing both groups, the average age of the FC Group was higher than the NFC group, and according to review studies the incidence of Alzheimer Disease rises with advancing age.^25-27^ Gender analysis showed that in the FC Group there was a high prevalence of females (75%) with a practically similar pattern in the NFC Group. Several other studies have shown a predominance of women with AD,^28,29^ but according to the authors concerned, this gender difference could have been due to longevity of women and not to any specific risk factor linked to sex.^30^ Education was poor in both FC and NFC Group, showing that both groups were equivalent for schooling.

To complete the study and certify that reassessment was valid, MMSE, time in use of medication, prescribed doses and DAD in both groups were compared. Comparison of MMSE in both groups revealed that patients from the FC Group had lower scores than those from the NFC Group. Concerning medication use, patients from the FC Group used higher doses for a longer period. In this study, better performance was observed by patients in the Group NFC than in the FC Group on the activities evaluated in the DAD, although in both groups high values of standard deviation were found.

These data corroborate the hypothesis that there were indeed two distinct groups, with the second not meeting the diagnostic criteria, exhibiting much higher MMSE scores, sub therapeutic doses and a DAD performance closer to normal. As this was a pilot trial of a regional nature, there was probably some interference, a theory warranting further studies.

According to the results of this study, a great number of patients using anti-cholinesterase were observed that did not fulfill the diagnosis criteria for AD, thereby resulting in a great negative impact both for the patient, his/her family as well as society.

When the two groups were compared, different behaviour between them was observed, thus corroborating the hypothesis of inadequate inclusion of patients in the NFC under the Exceptional Drugs Program.

As AD diagnosis is based on memory related deficits, the NFC Group may have included patients at initial phases of the disease and also those with other diagnoses such as age-related cognitive deficit, vascular dementia, depression and other psychiatric manifestations, where systematized studies and follow-ups of these patients would help characterize the underlying disease.

Due to the symptomatology dynamics of the disease, while on the one hand treatment may be postponed with anti cholinesterases, on the other, patients may be precociously introduced into taking the drug. The results of this study suggest that more comprehensive criteria should be adopted in the inclusion of patients onto the Exceptional Drugs Program.

In short, it has been concluded that in the dispensation of medications, 51% of patients did not fulfill the AD diagnostic criteria, a finding reinforced on analysis of the variables MMSE, dose, time in use of medication and scores on the DAD.
